# Beyond Rare-Variant Association Testing: Pinpointing Rare Causal Variants in Case-Control Sequencing Study

**DOI:** 10.1038/srep21824

**Published:** 2016-02-23

**Authors:** Wan-Yu Lin

**Affiliations:** 1 Institute of Epidemiology and Preventive Medicine, College of Public Health, National Taiwan University, Taipei, Taiwan; 2 Department of Public Health, College of Public Health, National Taiwan University, Taipei, Taiwan

## Abstract

Rare-variant association testing usually requires some method of aggregation. The next important step is to pinpoint individual rare causal variants among a large number of variants within a genetic region. Recently Ionita-Laza *et al.* propose a backward elimination (BE) procedure that can identify individual causal variants among the many variants in a gene. The BE procedure removes a variant if excluding this variant can lead to a smaller *P*-value for the BURDEN test (referred to as “BE-BURDEN”) or the SKAT test (referred to as “BE-SKAT”). We here use the adaptive combination of *P*-values (ADA) method to pinpoint causal variants. Unlike most gene-based association tests, the ADA statistic is built upon per-site *P*-values of individual variants. It is straightforward to select important variants given the optimal *P*-value truncation threshold found by ADA. We performed comprehensive simulations to compare ADA with BE-SKAT and BE-BURDEN. Ranking these three approaches according to positive predictive values (PPVs), the percentage of truly causal variants among the total selected variants, we found ADA > BE-SKAT > BE-BURDEN across all simulation scenarios. We therefore recommend using ADA to pinpoint plausible rare causal variants in a gene.

Next-generation sequencing (NGS) technologies enable the measurement of epigenetic information for the entire genome at a high resolution^1^,^2^,^3^,^4^. Due to the extremely low minor allele frequencies (MAFs), detecting individual rare causal variants is difficult. To strengthen signals, most statistical methods test the combined effects of rare variants in a gene or a functional unit^5^,^6^,^7^,^8^,^9^,^10^,^11^,^12^,^13^,^14^,^15^,^16^,^17^,^18^,^19^,^20^,^21^,^22^,^23^,^24^,^25^. These statistical methods can be classified into three categories: (1) the BURDEN test^5^,^6^,^7^,^8^; (2) the sequence kernel association test (SKAT)^10^,^11^,^12^; and (3) the *P*-values combination methods^13^,^14^,^15^,^16^,^26^.

The BURDEN test is more powerful than SKAT when the proportion of causal variants in a region is large and all causal variants are deleterious/protective^13^,^14^,^27^,^28^. SKAT, however, is superior to the BURDEN test when the number of neutral variants increases and/or both deleterious and protective variants coexist in a gene^28^. Moreover, because many neutral variants may be included in an NGS analysis, it is worthwhile to truncate variants with larger *P*-values that are more likely to be neutral^13^,^26^,^29^. With this concept, one of the *P*-values combination methods^13^,^14^,^15^,^16^,^26^, the “adaptive combination of *P*-values method” (abbreviated as “ADA”)^13^, is applicable to NGS data analyses.

Sequencing a gene in thousands of subjects can detect hundreds of rare variants^30^. Each of the above gene-based methods reports a *P*-value for the association of multiple rare variants and the disease. However, the following identification of a small proportion of truly causal variants is an even more difficult challenge. The BURDEN tests and SKAT group the variants in a gene to form statistics, but it is not easy to pinpoint individual causal variants from the composite statistics. Recently, Ionita-Laza *et al.* propose a backward elimination (BE) procedure to identify individual causal variants^30^. The BE procedure removes a variant if excluding it can lead to a smaller *P*-value for the BURDEN test (referred to as “BE-BURDEN”) or the SKAT test (referred to as “BE-SKAT”).

The BE algorithm determines the number of interesting variants by applying the backward elimination to the entire list of variants. Then a resampling procedure is used to select interesting variants, with the following four steps:

(1) Randomly sampling *r* (say, *r* = 20) variants from the region of interest, to form a current set (denoted by 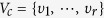). Computing the *P*-value of SKAT (or BURDEN) test with the variants in the current set, denoted by 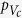.

(2) Removing each of the variants one at a time from 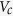 and computing the *P*-value of SKAT (or BURDEN) test with the remaining variants, denoted by 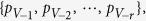 where 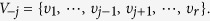


(3) Removing the *k*th variant from 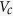 if 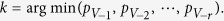 and if 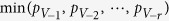

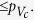
 Updating 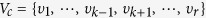 and computing a new 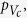 the *P*-value of SKAT (or BURDEN) test with the variants in the new current set 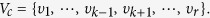


(4) Repeating steps (2) and (3) until 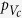 cannot be even smaller by removing any one variant from 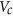. Then returning all the variants in 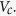


The above algorithm is applied to *B* (say, *B* = 1000) random subsamples. With B subsamples, Ionita-Laza *et al.* calculate the number of times each variant is returned in Step 4, and call this number the *return count* of a variant^30^. Then a nonparametric EM-like method^31^ is used to partition the variants into “interesting” (higher return counts) and “non-interesting” (lower return counts) groups.

In this work, we pinpoint rare causal variants via the ADA method^13^. Although ADA also aggregates association signals of multiple variants in a gene, its statistic is built upon per-site *P*-values of individual variants. Therefore, it is straightforward to pinpoint plausible causal variants via ADA. With extensive simulations, we compare the numbers of true positives (TPs) and false positives (FPs), and the positive predictive values (PPVs) of ADA and the two BE procedures. The three methods (ADA, BE-SKAT, and BE-BURDEN) are also applied to the Dallas Heart Study data^3^,^32^.

Because the previous ADA approach^13^ is directly applicable to pinpoint individual causal variants, here we use the same name (ADA) for the selection of causal variants. However, the previous SKAT 10,11 and BURDEN 5,6,7,8 tests cannot be directly used to identify individual causal variants. We therefore leave “SKAT” and “BURDEN” to be the names of tests, and let “BE-SKAT” and “BE-BURDEN” be the backward elimination procedures for the identification of individual causal variants.

## Results

### Simulation Study

We simulate 10,000 chromosomes of 5 kb (kilo base pairs), 10 kb, or 20 kb regions with a coalescent model^33^. The sequences were generated according to the linkage disequilibrium (LD) patterns of Europeans. We defined an analysis marker set that contained all variants with population MAF < 5%. This is a conventional MAF cutoff value to prohibit common variants from dominating the results of groupwise association tests for a gene. We specified 25% and 50% of rare variants (with MAF < 1%) as causal variants, respectively. Although 25% and 50% were not small percentages, many causal variants were not observed in a simulated sample that contained 500 cases and 500 controls, because of their low MAFs. After summarizing all simulated data sets, the causal percentages were found to be ~7.3% and ~14.6% in the analysis marker sets (all markers with MAF < 5%), respectively.

Table 1 lists the details of each simulation scenario (region length: 5 kb, 10 kb, or 20 kb; causal percentage: ~7.3%, or ~14.6%). The population attributable risk fraction (PAF) of each causal variant was set to be 0.3% and 0.5%, respectively. We let “*r*_*isk*_” be the percentage of risk variants from among the total causal variants, and “*r*_*isk*_” was specified as 5%, 20%, 50%, 80%, and 100%, respectively. When the region length was 5 kb, the number of causal variants was small (see Table 1) so that specifying *r*_*isk*_ as five levels made no sense. In this situation, we let *r*_*isk*_ be 0%, 50%, and 100%, respectively. Given *r*_*isk*_, the number of risk variants was #(causal variants) 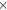 *r*_*isk*_. When this number was not an integer, we modified it to be 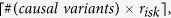 where 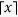 was the smallest integer not less than *x*.

According to the definition of PAF, we can obtain the relationship between it and the genotype relative risk (GRR)^34^. The GRR of a causal variant is 
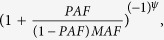
 where *MAF* is the population MAF of that variant, and 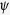 is an indicator variable (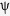 = 1 if that variant is protective; 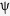 = 0 if deleterious). To form the genotypes of a subject, we randomly chose two haplotypes from the pool of 10,000 haplotypes. Following the simulation setting of previous association studies^13^,^35^,^36^,^37^, the disease status of a subject with haplotypes *H*_*1*_ and *H*_*2*_ is


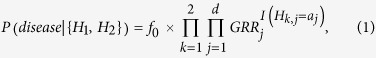


where 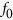 is the baseline penetrance, H_*k,j*_ is the allele at the *j*th causal variant site (*j* = 1, …, *d*, in which *d* is the number of causal variants) on the haplotype *H*_*k*_ (*k* = 1, 2), and 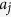 is the minor allele (served as causal allele) at the *j*th causal variant site. Throughout the simulation, we specified the baseline penetrance 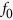 as 1%, and 500 cases and 500 controls were analyzed in each replication.

As pointed out by Wang *et al.*^38^, if properly modelled, the impact of a protective variant is minor compared to the impact of a deleterious variant. Our above simulation setting has been shown to assign a smaller effect size to a protective variant than to a deleterious variant with the same MAF^13^.

## Competitor Methods

We compared the number of TPs and FPs, and the PPVs of ADA^13^, BE-SKAT, and BE-BURDEN^30^. The ADA code was downloaded from http://homepage.ntu.edu.tw/~linwy/ADAprioritized.html and was implemented with 1,000 permutations. BE (for both BE-SKAT and BE-BURDEN) was downloaded from the authors,^30^ website http://www.columbia.edu/~ii2135/ and was conducted with 1,000 random subsamples.

Throughout this work (including simulations and the real data application), the *P*-value truncation thresholds considered in ADA were 0.10, 0.11, 0.12, …, and 0.20. Using a wider range of *P*-value truncation thresholds, say 0.05, 0.06, …, 0.25, will not contribute a noticeable power gain to ADA given a typical sequencing study sample size^13^. About the pre-specified weight given to the *j*th variant (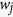), we followed the original SKAT paper^10^ to set 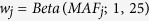, where 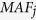 was the MAF (across cases and controls combined) of that variant. To have a fair comparison, we used this weighting scheme for all the three methods.

### Simulation Results

**Figure 1:**
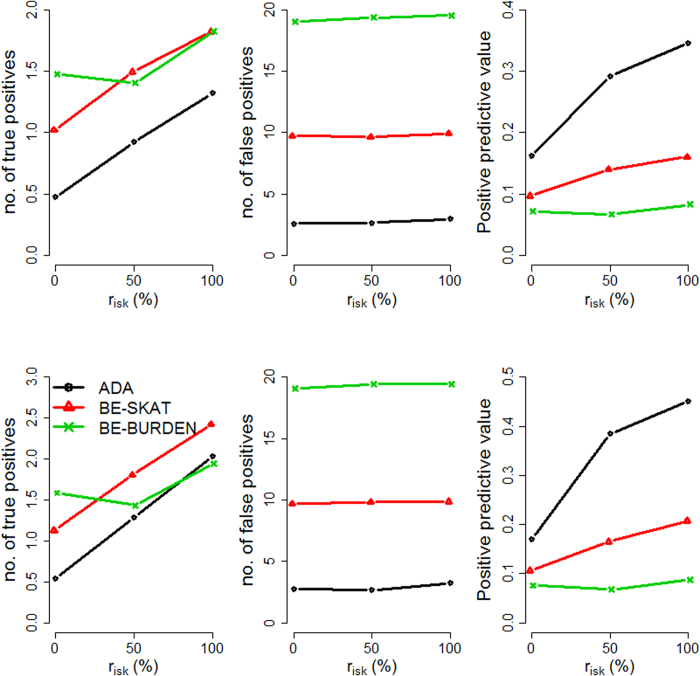
Results for 5 kb regions, given a causal percentage of ~7.3%. Top row: PAF = 0.3%; bottom row: PAF = 0.5%. The *x*-axis is the percentage of risk variants from among the total causal variants, whereas the *y*-axis is the mean number of true positives (left column), the mean number of false positives (middle column), or the mean positive predictive values (right column), based on 1,000 replications.

**Figure 2:**
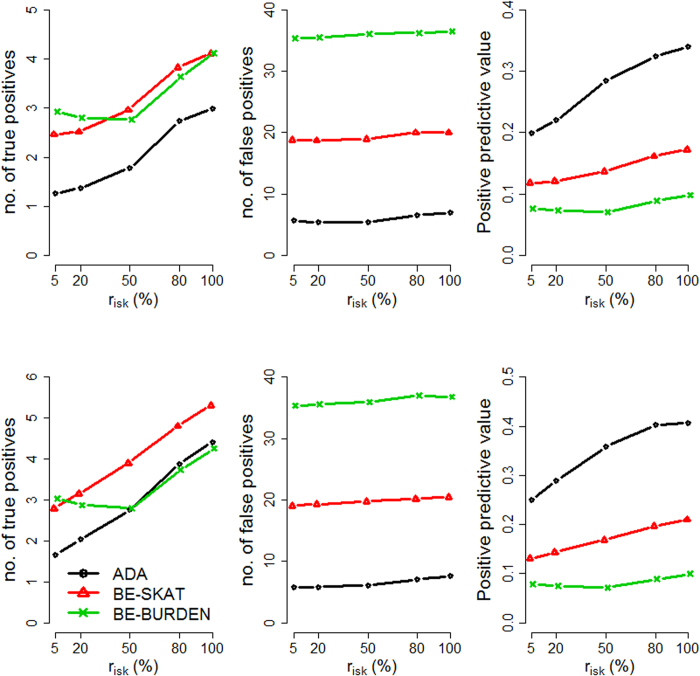
Results for 10 kb regions, given a causal percentage of ~7.3%. Top row: PAF = 0.3%; bottom row: PAF = 0.5%. The *x*-axis is the percentage of risk variants from among the total causal variants, whereas the *y*-axis is the mean number of true positives (left column), the mean number of false positives (middle column), or the mean positive predictive values (right column), based on 1,000 replications.

**Figure 3:**
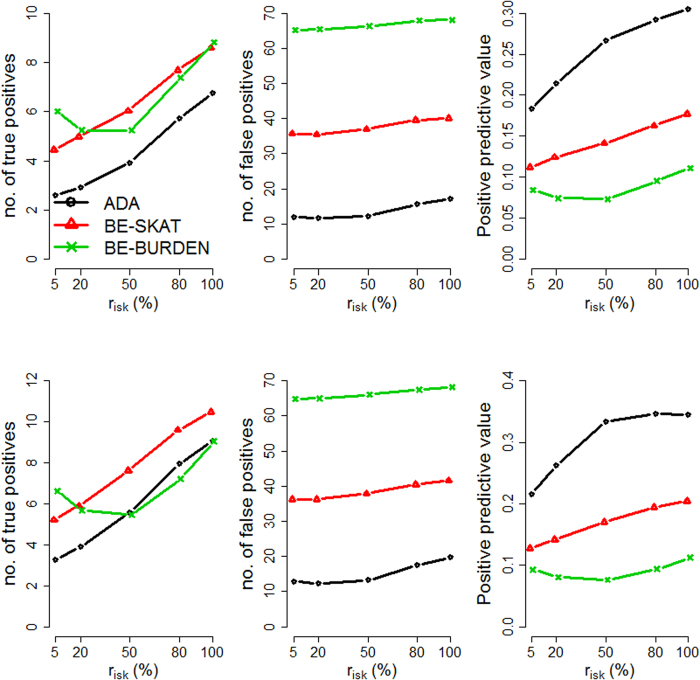
Results for 20 kb regions, given a causal percentage of ~7.3%. Top row: PAF = 0.3%; bottom row: PAF = 0.5%. The *x*-axis is the percentage of risk variants from among the total causal variants, whereas the *y*-axis is the mean number of true positives (left column), the mean number of false positives (middle column), or the mean positive predictive values (right column), based on 1,000 replications.

**Figure 4:**
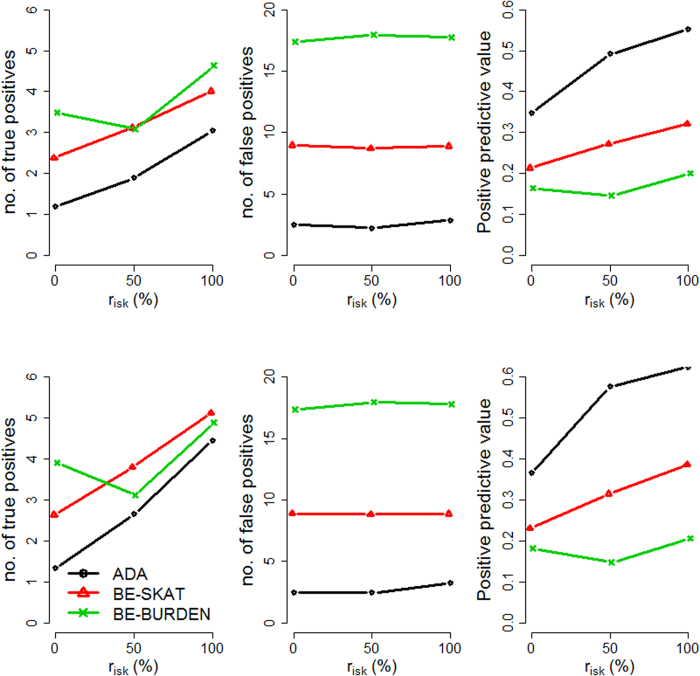
Results for 5 kb regions, given a causal percentage of ~14.6%. Top row: PAF = 0.3%; bottom row: PAF = 0.5%. The *x*-axis is the percentage of risk variants from among the total causal variants, whereas the *y*-axis is the mean number of true positives (left column), the mean number of false positives (middle column), or the mean positive predictive values (right column), based on 1,000 replications.

**Figure 5:**
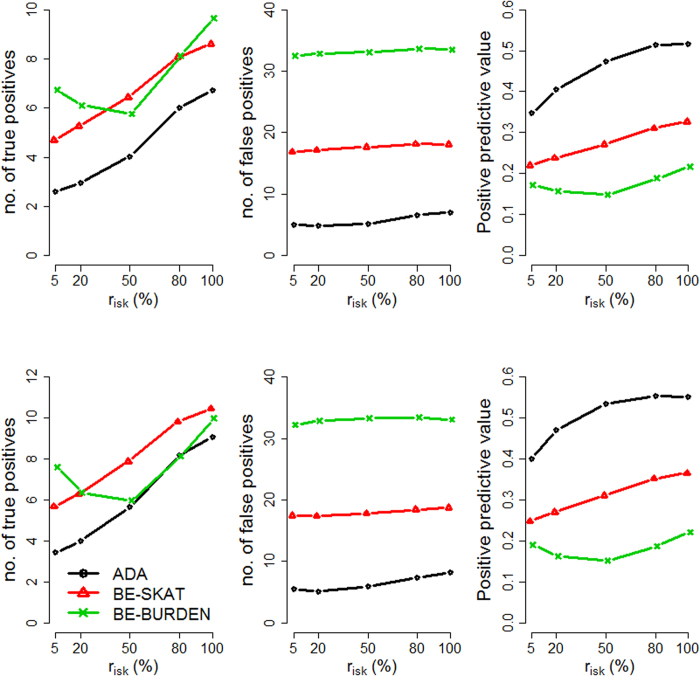
Results for 10 kb regions, given a causal percentage of ~14.6%. Top row: PAF = 0.3%; bottom row: PAF = 0.5%. The *x*-axis is the percentage of risk variants from among the total causal variants, whereas the *y*-axis is the mean number of true positives (left column), the mean number of false positives (middle column), or the mean positive predictive values (right column), based on 1,000 replications.

**Figure 6:**
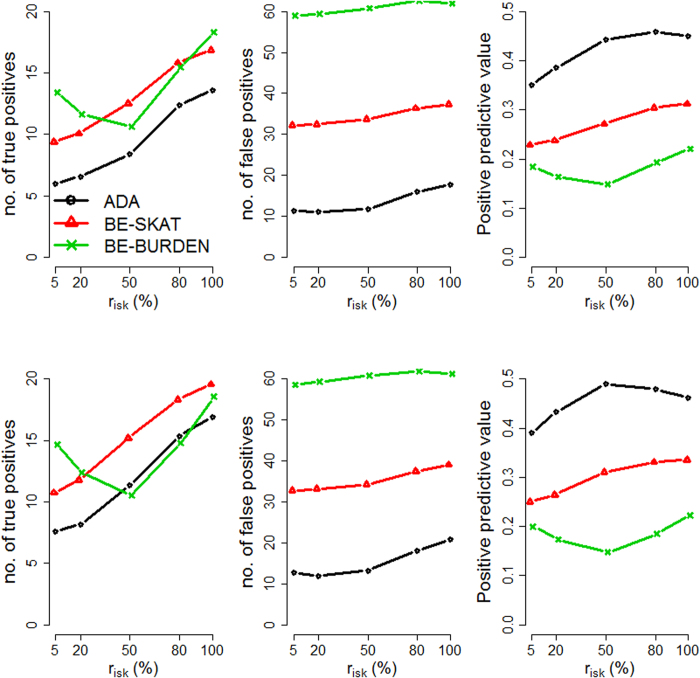
Results for 20 kb regions, given a causal percentage of ~14.6%. Top row: PAF = 0.3%; bottom row: PAF = 0.5%. The *x*-axis is the percentage of risk variants from among the total causal variants, whereas the *y*-axis is the mean number of true positives (left column), the mean number of false positives (middle column), or the mean positive predictive values (right column), based on 1,000 replications.

We evaluated the performance of the three methods by varying four factors: (1) causal percentage (the percentage of causal variants among all variants): lower (~7.3%, Figs 1, 2, 3) vs. higher (~14.6%, Figs 4, 5, 6); (2) region length: 5 kb (Figs 1 and 4), 10 kb (Figs 2 and 5), or 20 kb (Figs 3 and 6); (3) effect size of each causal variant: PAF = 0.3% (top rows of Figs 1, 2, 3, 4, 5, 6) vs. 0.5% (bottom rows of Figs 1, 2, 3, 4, 5, 6); (4) percentage of risk variants from among the total causal variants (*x*-axis in Figs 1, 2, 3, 4, 5, 6, “*r*_*isk*_”).

Let #(*TP*) and #(*FP*) be the numbers of TPs and FPs, respectively. PPV is defined as #(*TP*)/[#(*TP*) + #(*FP*)], which is the percentage of true positives out of all positives. That is, the percentage of truly causal variants among the total selected variants. In the following, we discuss the mean and variability in #(*TP*), #(*FP*), and PPVs, for the three methods.

### Mean performance in #(TP), #(FP), and PPVs

Figs 1, 2, 3 present the results for 5 kb, 10 kb, and 20 kb, respectively, given the causal percentage of ~7.3% and 1,000 replications. Among the three methods, ADA always provided the shortest list of important variants. BE-SKAT and BE-BURDEN detected more TPs than ADA, however they also yielded more FPs and smaller PPVs than ADA. BE-BURDEN generated the largest #(*FP*) and the smallest PPVs among all the three methods. The results given a higher causal percentage (~14.6%) and 1,000 replications were shown in Figs 4, 5, 6, which were quite similar to Figs 1, 2, 3. Among the three methods, ADA provided the largest PPV and the fewest FPs across all scenarios.

PPV is the percentage of truly causal variants among the total selected variants. Ranking the three approaches according to PPV, we have PPV_ADA_ > PPV_BE-SKAT_ > PPV_BE-BURDEN_ across all scenarios. From PPV_ADA_ > PPV_BE_, we have #(*TP*)_ADA_/[#(*TP*)_ADA_ + #(*FP*)_ADA_]> #(*TP*)_BE_/[#(*TP*)_BE_ + #(*FP*)_BE_]. This is equivalent to #(*TP*)_ADA_/#(*FP*)_ADA_ > #(*TP*)_BE_/#(*FP*)_BE_, meaning that the signal-to-noise ratio of ADA is larger than that of the BE approach. ADA has the smallest #(*FP*) so that the unnecessary cost on following investigation to false-positive variants can be decreased. Furthermore, its larger PPV represents a larger signal-to-noise ratio. However, if an investigator prefers a larger #(*TP*) to a larger PPV, he/she may choose the BE approach at the cost of more FPs.

In the following, we discuss the impact of the four factors accordingly:

### Causal percentage

As the causal percentage increased, #(*TP*) and PPVs increased, whereas #(*FP*) decreased. The relative performance of the three methods did not vary with the increasing causal percentage.

### Region length

Because the causal percentage was fixed from Figs 1, 2, 3 (or from Figs 4–6), #(*TP*) and #(*FP*) increased at roughly the same rate as the increase in region length. PPVs did not change much with the different region lengths. The relative performance of the three methods remained the same across the three region lengths.

### Effect size of each causal variant

With a larger effect size, #(*TP*) increased while #(*FP*) remained unchanged, and therefore PPVs increased. When the PAF associated with each causal variant was increased from 0.3% to 0.5%, ADA and BE-SKAT had larger improvements in mean #(*TP*) than BE-BURDEN did. This is because, for ADA, the identification of variants relies on per-site *P*-values of individual variants. The PAF of 0.3% (corresponding to GRR ≈ 1.3 for a 1% deleterious variant) was too small for ADA to identify more TPs. When the PAF was enlarged to 0.5% (corresponding to GRR ≈ 1.5 for a 1% deleterious variant), more causal variants could be pinpointed according to their per-site *P*-values. For BE-SKAT, its larger improvement in #(*TP*) can be traced back to the statistic of the SKAT test. The SKAT statistic is a weighted sum of squared single-variant score statistics^39^. When the PAF was enlarged to 0.5%, causal variants had larger squared single-variant score statistics and were more easily to be detected.

### Percentage of risk variants from among the total causal variants

As the proportion of risk variants increased, BE-SKAT and ADA identified more TPs. However, BE-BURDEN had a V-shaped curve in the sense that the minimum #(*TP*) occurred around 50% risk variants and 50% protective variants. To explain this, we compare the statistics of the SKAT and BURDEN tests. The SKAT statistic is a weighted sum of squared single-variant score statistics^39^. Because risk variants had larger effect sizes than protective variants with similar MAFs^13^,^38^, the squared single-variant score statistics of individual risk variants made greater contributions to the SKAT statistic than protective variants did. Therefore, as the proportion of risk variants increased, BE-SKAT identified more TPs. ADA identified causal variants according to per-site *P*-values. Risk variants had larger effect sizes^13^,^38^, corresponding to smaller per-site *P*-values, than protective variants. As the number of risk variants increased, more TPs with small per-site *P*-values could be found by ADA.

Things were different in BE-BURDEN. The BURDEN statistic is the square of a weighted sum of single-variant score statistics^39^. When a region included 50% risk variants and 50% protective variants, the single-variant score statistics contributed by risk variants were diluted by protective variants. Using the BURDEN statistic to detect the association of a gene/region was not appropriate in this scenario, and evaluating the contribution of each variant to this BURDEN statistic made no much sense. Therefore, it is not surprising that BE-BURDEN found fewer TPs in the presence of both risk and protective variants.

### Variability in #(*TP*), #(*FP*), and PPVs

The standard deviations of #(*TP*), #(*FP*), and PPVs were listed in Supplementary Tables S1–S3. A larger mean usually corresponded to a larger standard deviation. We therefore also listed the coefficients of variation (C.V., defined as the ratio of standard deviation to the mean) of #(*TP*), #(*FP*), and PPVs in Supplementary Tables S4–S6. Because the identification of variants relies on per-site *P*-values of individual variants, ADA generally had larger variability compared with its two competitors.

### Computation Time

**Figure 7:**
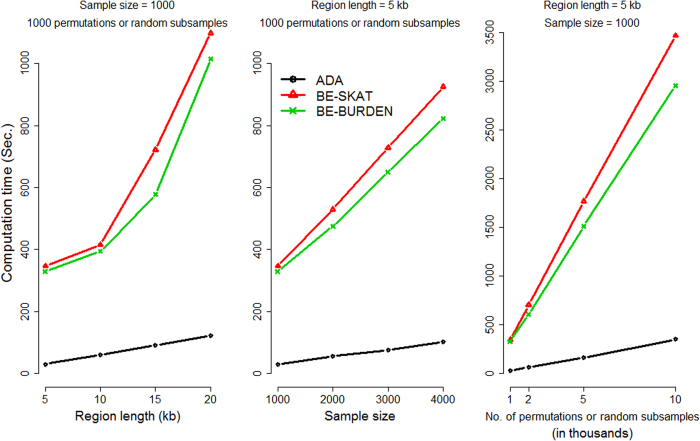
The mean computation time (in seconds) of every method, by varying the region length, the sample size, or the number of permutations (for ADA) or random subsamples (for BE-SKAT and BE-BURDEN). Left column: sample size =1000, the number of permutations or random subsamples =1000; middle column: region length =5 kb, the number of permutations or random subsamples =1000; right column: region length =5 kb, sample size =1000.

The computation time for the three methods depends on three factors: (1) the number of variants, (2) the sample size, and (3) the number of permutations (for ADA) or random subsamples (for BE-SKAT and BE-BURDEN). Figure 7 presents the mean computation time (in seconds) of every method, under several levels of the region length, the sample size, and the number of permutations or random subsamples. The required time was measured on a Linux platform with an Intel Xeon E5-2690 2.9 GHz processor and 4 GB memory. ADA is the most computationally efficient method, with a time complexity 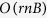, where *r* is the number of variants, *n* is the sample size, and *B* is the number of permutations. The BE procedure evaluates the contribution of each individual variants to the groupwise association test statistics (BURDEN or SKAT) and sequentially removes variants from the variant set. This step-by-step procedure requires more time than ADA.

### Application to the Dallas Heart Study Data

We then applied the three methods to the Dallas Heart Study (DHS) data. Romeo *et al.* sequenced seven exons and intron-exon boundaries of the *angiopoietin-like 4* (*ANGPTL4*) gene in 3,551 participants of DHS, in order to uncover the effects of genetic variants on human triglycerides^3^,^32^. In our analysis, we selected 1,045 European Americans from among the 3,551 DHS participants. Following the analysis in the DHS paper^32^, we stratified the 1,045 European Americans by sex (500 males and 545 females), and then compared the numbers of variants in the top and bottom quartiles of the triglyceride distribution. Wu *et al.*^10^ also analyzed this dichotomized phenotype on the highest and the lowest quartiles of each of the sex groups. In the male group, the 25^th^ and 75^th^ percentiles were 81 mg/dl and 187 mg/dl, respectively. In the female group, the 25^th^ and 75^th^ percentiles were 71 mg/dl and 152 mg/dl, respectively. Therefore, we treated 126 males with triglycerides 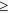
187 mg/dl and 137 females with triglycerides 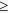
152 mg/dl as cases and 130 males with triglycerides 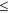
81 mg/dl and 138 females with triglycerides 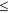 71 mg/dl as controls. In total, we had 263 cases and 268 controls.

Similar to our simulation study, we defined an analysis marker set to contain all variants with MAF < 5%. A synonymous variant at 8336810 bp was excluded because of a high missing rate (14.3%). Moreover, 65 cases and 66 controls with any missing genotypes in the test region were removed. Finally, the data set contained 198 cases and 202 controls. Table 2 lists the 17 genetic variants (inside the *ANGPTL4* gene) observed in this sample. We listed the numbers of variant carriers in cases/controls in Table 2. Because these 17 variants were rare or low-frequency (MAF < 5%), we did not observe any subject with homozygous minor alleles at any locus.

### Gene-based association tests

The *P*-values of the ADA, SKAT, and BURDEN methods for testing the association between triglycerides and the region containing these 17 loci were 0.0467, 0.0123, and 0.6962, respectively. The ADA test was implemented with the R code from http://homepage.ntu.edu.tw/~linwy/ADAprioritized.html, which could not only provide the *P*-value of the ADA gene-based association test but also pinpoint individual rare variants. Same as the previous simulation studies, the *P*-value truncation thresholds used in ADA were 0.10, 0.11, 0.12, …, and 0.20. The SKAT and BURDEN tests were performed with the SKAT package (version 1.1.2)^40^. Within the SKAT function, we specified the parameter r.corr (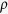) as 0 and 1 to obtain the *P*-values of the SKAT and BURDEN tests, respectively. Following the original SKAT paper^10^, the weight given to the *j*th variant (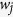) was set to be 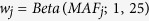, where 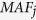 was the MAF (across cases and controls combined) of that variant. With a significance level of 0.05, only SKAT and ADA suggested an association of the *ANGPTL4* gene with triglycerides.

### Pinpointing individual rare variants

We then used the BE package^30^ (for both BE-SKAT and BE-BURDEN) (http://www.columbia.edu/~ii2135/) to pinpoint individual rare variants. Variants pinpointed by ADA, BE-SKAT, or BE-BURDEN, were marked in Table 2. ADA found only one important variant: E40K, which was also selected by BE-SKAT. E40K is a rare variant, reported to have a MAF of ~1.3% in European Americans^32^,^41^. It was previously reported to be associated with significantly lower plasma levels of triglyceride in European Americans^32^,^41^,^42^,^43^,^44^. BE-SKAT and BE-BURDEN pinpointed three and seven important variants, respectively.

To the best of our knowledge, among the 17 variants listed in Table 2, only E40K has been reported to be associated with plasma levels of triglyceride in European Americans^32^,^41^,^42^,^43^,^44^. ADA only pinpointed this variant, which was consistent with our simulation result that ADA always selected the fewest variants among the three methods. BE-SKAT pinpointed two more variants than ADA. However, these two additional variants have not been reported to be associated with triglycerides. Based on our simulation results, BE-SKAT selected more FPs than ADA. We have to be more cautious with these two variants. BE-BURDEN pinpointed seven variants. Because the BURDEN test did not suggest a significant association between triglycerides and the region containing these 17 loci (*P*-value* *= 0.6962), there was no need to investigate these seven variants in details.

## Discussion

Single marker approaches are under-powered for sequencing studies with typical sample sizes, and therefore rare-variant association testing usually requires some strategy of aggregation^45^. The next important step is to identify individual rare causal variants from the promising regions/genes. Identifying a small number of rare causal variants that contribute to complex diseases has become a major focus of investigation^46^. We here recommend using ADA to pinpoint important rare variants that may be responsible for the disease pathogenesis. We compare ADA with the BE procedure based on the BURDEN test or the SKAT test^30^. This work is not a power comparison between ADA, BURDEN, and SKAT–which has been addressed in a previous study^13^. ADA can be more powerful than BURDEN and SKAT, because it truncates variants with larger *P*-values that are more likely to be neutral. This purification of association signals can enhance the statistical power of a gene-based test.

In this work, we focus on identifying rare causal variants from among the variants in a gene, instead of testing the significance for a group of variants. To have a pure evaluation of the performance to identify rare causal variants, we did not assess the association for a group of variants with the ADA, SKAT, or BURDEN test before pinpointing individual variants. The results shown in Figs 1, 2, 3, 4, 5, 6 were not filtered by the significance of ADA, SKAT, or BURDEN.

However, in practice, it is not reasonable to go to the step of pinpointing individual variants, if gene-based association tests are not statistically significant. We therefore also show the results with consideration of gene-based association testing. Supplementary Figs S1–S3 present the mean #(*TP*), #(*FP*), and PPVs, of the extra simulations. In these figures we show the mean #(*TP*) (or #(*FP*), PPVs) based on all replications (regardless of the significance of ADA, SKAT, or BURDEN), and that based on the replications with *P*-values < 0.001 (or 0.0001) in the corresponding association tests. The association signals within a region, including both true association signals from causal variants and false association signals from neutral variants, are more significant, when the *P*-value of the corresponding association test is smaller. Therefore, we see larger means in #(*TP*) and #(*FP*) given more significant test results. The increase in #(*TP*) or #(*FP*) is larger when all causal variants are protective than when they are deleterious. This is because protective variants have smaller effect sizes than deleterious variants with similar MAFs^13^,^38^. If the association test for a gene containing protective variants can reach a stringent significance threshold (say, 0.0001), presumably there are more true signals from causal variants and/or more false signals from neutral variants. Therefore, it is not surprising that the mean #(*TP*) and #(*FP*) have a larger increase at *r*_*isk*_* *= 0% than at *r*_*isk*_ = 100%. The mean PPVs generally have a slight increase given more significant test results. The relative performance of the three methods remains the same as that shown in our simulation study, where the results were not filtered by the significance of ADA, SKAT, or BURDEN.

Moreover, we also studied the situations where a region actually contained no causal variants. Table 3 lists the mean #(*FP*), and the corresponding standard deviation, when the region in fact includes no causal variant. FPs from ADA were fewer, compared with its two competitors. Consistent with the aforementioned simulation results, BE-BURDEN generated the most FPs. In this situation that all variants were neutral, the association signals were actually all false alarms from neutral variants. Therefore, it is not surprising that a larger mean #(*FP*) can be found in replications with smaller *P*-values in association tests.

We find that ADA greatly enhances PPVs compared with the BE procedure based on BURDEN or SKAT^30^. Furthermore, ADA is more computationally feasible than the two competitors. We therefore recommend using ADA to pinpoint plausible rare causal variants in a gene.

## Methods

### Adaptive Combination of P-values (ADA) Algorithm to Pinpoint Rare Causal Variants from A Large Number of Variants in A Gene

Given *r* variants in a gene of interest, *J* candidate *P*-value truncation thresholds (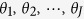), pre-specified weights for variants (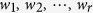), and a significance level for gene-based association tests, the ADA algorithm is performed as follows:

**Step 1.** Obtain per-site *P*-values for the *r* variants, 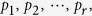, using appropriate single-variant tests. For binary trait analysis without confounder adjustment, per-site *P*-values can be obtained by the Fisher’s exact test^47^. To adjust confounders, logistic (linear) regression model can be used in analyses for binary (quantitative) traits.

Following Cheung *et al.*^15^, in this work we used the Fisher’s exact test^47^ to assess the significance of each variant. For a variant that is more frequent in cases than in controls, we let *X* be the random variable representing the number of minor-allele counts in the case group, 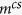 be the observed minor-allele counts in the case group, 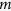 be the total minor-allele counts in the case and control groups, 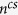 be the observed number of alleles in the case group, and *n* be the total number of alleles in the case and control groups. The mid-*P*-value for the association of this variant with the disease is


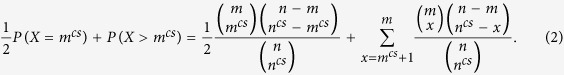


**Step 2.** Under the *j*th *P*-value truncation threshold, calculate two significance scores, 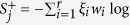

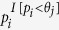
 and 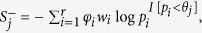 where 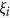 is 1 if the *i*th variant is ‘deleterious-inclined’ (with larger variant frequencies in cases than in controls) and 0 otherwise, 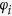 is 1 if the *i*th variant is ‘protective-inclined’ (with larger variant frequencies in controls than in cases) and 0 otherwise, and 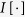 is an indicator variable with two possible values: 0 and 1.

**Step 3.** Across the *J P*-value truncation thresholds, obtain 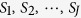, where 
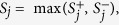
 for *j* = 1, 2, …, *J*. With *B* permutations, the *P*-value of 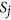 is estimated by 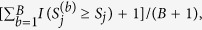 where 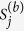 is calculated with the *b*th permuted sample under the *j*th *P*-value truncation threshold, *b *= 1, …, *B*. The *P*-value of 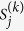 is estimated by 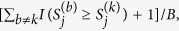 *k* = 1, …, *B*. For the observed sample and the *b*th permuted sample, the minimum *P*-values across the *J P*-value truncation thresholds are denoted by 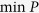 and 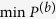, respectively. Then the ‘final *P*-value’ is 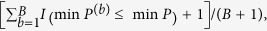 which is the *P*-value of the ADA test^13^ for the association of a gene/region with the disease. If this ‘final *P*-value’ is larger than the pre-specified significance level, stop here. We conclude that the gene/region is not associated with the disease.

**Step 4.** If the ‘final *P*-value’ is smaller than the significance level, select all variants with per-site *P*-values smaller than the optimal *P*-value truncation threshold, i.e., the *P*-value threshold producing the minimum *P*-value for the unpermuted sample.The R code to implement the ADA method can be downloaded from http://homepage.ntu.edu.tw/~linwy/ADAprioritized.html. The above Steps 1–3 are used to perform the ADA test^13^. By adding Step 4, ADA is directly applied to the selection of individual causal variants.

## Additional Information

**How to cite this article**: Lin, W.-Y. Beyond Rare-Variant Association Testing: Pinpointing Rare Causal Variants in Case-Control Sequencing Study. *Sci. Rep.* **6**, 21824; doi: 10.1038/srep21824 (2016).

## Supplementary Material

Supplementary Information

## Figures and Tables

**Table 1:** The details of each simulation scenario.

Causal percentage^*a*^	Region length	No. of variant sites (mean)	No. of causal variants (mean)	Results
Lower (~7.3%)	5 kb	~45	~3	Fig. 1
	10 kb	~91	~7	Fig. 2
	20 kb	~182	~14	Fig. 3
Higher (~14.6%)	5 kb	~45	~6	Fig. 4
	10 kb	~91	~14	Fig. 5
	20 kb	~182	~28	Fig. 6

^a^Causal percentage: The percentage of causal variants from among all the variants in the analysis marker set.

**Table 2:** Association analysis for the *ANGPTL4* gene and triglycerides.

Genomic position (bp)^32^	Variant name^32^	Pinpointed by the method (marked by ‘V’)			Non-synonymous(marked by ‘Y’)	No. of variant carriers in cases^c^	No. of variant carriers in controls^c^
		ADA^a^	BE-SKAT^b^	BE-BURDEN^b^			
8335323	E40K	V	V		Y	1	8
8337000			V	V		2	0
8337027				V		1	0
8337155	E167K				Y	0	1
8337250						0	1
8340030						0	1
8340185	P210P					1	0
8340204	K217X				Y	0	1
8341802				V		1	0
8341945	G223R				Y	0	1
8342029	P251T			V	Y	1	0
8342288	P307P					1	0
8342289	V308M			V	Y	1	0
8342373	R336C				Y	1	3
8342438			V	V		2	0
8344630	G361S				Y	0	1
8344771				V		1	0
Total pinpointed causal variants		1	3	7			

^a^The ADA method was implemented with 1,000 permutations.

^b^The BE procedure (for both SKAT and BURDEN) was implemented with 1,000 random subsamples.

^c^Variant carriers were heterozygotes for all the 17 loci. We did not observe any subject with homozygous minor alleles at any locus.

**Table 3:** The mean number of false positives (and the corresponding standard deviation) [the number of replications considered] when the region actually contains no causal variant^a^.

*P*-value of ADA, SKAT, or BURDEN test	ADA^b^	BE-SKAT^c^	BE-BURDEN^c^
No restriction (all results)	2.92 (2.37) [10000]	10.57 (3.92) [10000]	20.66 (4.10) [10000]
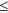 0.05	6.01 (3.24) [496]	12.54 (3.85) [510]	24.24 (4.67) [495]
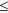 0.01	6.78 (3.62) [92]	12.89 (3.72) [108]	25.29 (4.03) [102]
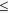 0.001	12.33 (6.66) [7]	12.89 (3.95) [9]	25.00 (5.00) [9]

^a^Region length =5 kb; causal percentage =0%; no. of cases=no. of controls =500; the total number of replications =10000

^b^The ADA method was implemented with 10,000 permutations.

^c^Due to a longer computation time, the BE procedure (for both BE-SKAT and BE-BURDEN) was implemented with 1,000 random subsamples.
