# Supplementary material for: Beyond Rare-Variant Association Testing: Pinpointing Rare Causal Variants in Case-Control Sequencing Study
Source: Sci Rep. 2016 Feb 23;6:21824. doi: 10.1038/srep21824 (PMC4763184; doi:10.1038/srep21824)
Supplement: Supplementary Information [file srep21824-s1.pdf]

Beyond Rare-Variant Association Testing: Pinpointing Rare Causal Variants in Case-Control Sequencing Study

Wan-Yu Lin

5 kb region length	Standard deviation of no. of true positives			Standard deviation of no. of false positives			Standard deviation of positive predictive value		
	r_{isk} =0%	r_{isk} =50%	r_{isk} =100%	r_{isk} =0%	r_{isk} =50%	r_{isk} =100%	r_{isk} =0%	r_{isk} =50%	r_{isk} =100%
**Causal percentage of ~7.3%, PAF = 0.3% (referred to the top row of Fig. 1)**									
ADA	0.71	0.89	1.11	2.24	2.35	2.63	0.25	0.29	0.28
BE-SKAT	0.91	1.02	1.17	3.67	3.84	3.89	0.09	0.10	0.10
BE-BURDEN	1.17	0.93	1.53	4.00	4.01	4.14	0.06	0.04	0.07
**Causal percentage of ~7.3%, PAF = 0.5% (referred to the bottom row of Fig. 1)**									
ADA	0.76	0.93	1.20	2.33	2.31	2.95	0.25	0.30	0.26
BE-SKAT	0.98	1.03	1.22	3.69	3.91	4.05	0.10	0.10	0.11
BE-BURDEN	1.31	0.89	1.70	3.89	3.99	4.12	0.06	0.04	0.07
**Causal percentage of ~14.6%, PAF = 0.3% (referred to the top row of Fig. 4)**									
ADA	1.34	1.49	1.90	2.30	2.09	2.47	0.32	0.31	0.26
BE-SKAT	1.51	1.64	1.94	3.65	3.59	3.80	0.13	0.14	0.14
BE-BURDEN	2.13	1.57	2.90	3.79	3.89	4.07	0.10	0.07	0.12
**Causal percentage of ~14.6%, PAF = 0.5% (referred to the bottom row of Fig. 4)**									
ADA	1.38	1.58	2.06	2.17	2.34	2.63	0.31	0.26	0.21
BE-SKAT	1.55	1.71	2.06	3.47	3.76	4.13	0.12	0.14	0.15
BE-BURDEN	2.30	1.49	3.29	3.95	3.86	4.08	0.10	0.06	0.13

Supplementary Table S1 Standard deviations of the numbers of true positives and false positives, and positive predictive values (when region length = 5 kb)

10 kb region length	Standard deviation of no. of true positives					Standard deviation of no. of false positives					Standard deviation of positive predictive value				
r_{isk}	5%	20%	50%	80%	100%	5%	20%	50%	80%	100%	5%	20%	50%	80%	100%
**Causal percentage of ~7.3%, PAF = 0.3% (referred to the top row of Fig. 2)**															
ADA	1.23	1.21	1.29	1.78	1.75	4.16	4.15	3.92	4.94	4.97	0.20	0.20	0.22	0.19	0.19
BE-SKAT	1.43	1.37	1.53	1.84	1.79	5.32	5.59	5.55	5.99	6.04	0.07	0.06	0.07	0.07	0.07
BE-BURDEN	1.70	1.45	1.35	2.34	2.80	5.84	5.76	5.84	6.10	6.39	0.04	0.04	0.03	0.05	0.06
**Causal percentage of ~7.3%, PAF = 0.5% (referred to the bottom row of Fig. 2)**															
ADA	1.33	1.36	1.58	1.75	1.84	4.01	3.99	4.49	5.03	4.98	0.20	0.20	0.20	0.18	0.17
BE-SKAT	1.41	1.40	1.60	1.73	1.77	5.36	5.54	5.88	6.04	5.81	0.06	0.06	0.06	0.07	0.07
BE-BURDEN	1.76	1.37	1.37	2.61	3.28	5.74	6.06	5.72	6.10	6.03	0.04	0.03	0.03	0.06	0.07
**Causal percentage of ~14.6%, PAF = 0.3% (referred to the top row of Fig. 5)**															
ADA	2.26	2.21	2.45	3.01	3.00	3.87	3.70	3.94	4.71	4.32	0.23	0.22	0.20	0.18	0.16
BE-SKAT	2.09	2.23	2.54	3.00	2.94	5.00	5.03	5.56	5.81	5.44	0.09	0.09	0.09	0.09	0.09
BE-BURDEN	2.93	2.48	2.25	4.43	5.06	5.96	5.82	5.49	5.75	6.03	0.07	0.06	0.05	0.09	0.11
**Causal percentage of ~14.6%, PAF = 0.5% (referred to the bottom row of Fig. 5)**															
ADA	2.42	2.50	2.53	2.98	2.89	3.69	3.77	4.33	4.40	4.43	0.21	0.20	0.18	0.15	0.14
BE-SKAT	2.36	2.30	2.73	3.00	2.96	5.08	5.35	5.48	5.46	5.82	0.09	0.09	0.09	0.09	0.09
BE-BURDEN	3.26	2.60	2.27	5.04	6.00	5.99	5.98	5.80	5.71	6.12	0.08	0.06	0.05	0.11	0.13

Supplementary Table S2 Standard deviations of the numbers of true positives and false positives, and positive predictive values (when region length = 10 kb)

20 kb region length	Standard deviation of no. of true positives					Standard deviation of no. of false positives					Standard deviation of positive predictive value				
r_{isk}	5%	20%	50%	80%	100%	5%	20%	50%	80%	100%	5%	20%	50%	80%	100%
**Causal percentage of ~7.3%, PAF = 0.3% (referred to the top row of Fig. 3)**															
ADA	2.10	2.00	2.34	2.83	2.97	7.21	7.20	7.68	9.32	9.59	0.12	0.12	0.14	0.12	0.12
BE-SKAT	1.99	2.06	2.45	2.74	2.88	8.22	8.31	9.08	9.61	10.01	0.05	0.05	0.05	0.05	0.05
BE-BURDEN	2.71	2.15	2.13	4.17	5.18	8.89	9.69	9.63	10.00	10.50	0.04	0.03	0.03	0.05	0.06
**Causal percentage of ~7.3%, PAF = 0.5% (referred to the bottom row of Fig. 3)**															
ADA	2.38	2.33	2.60	2.76	2.82	8.05	7.75	8.71	9.61	10.07	0.13	0.13	0.14	0.13	0.12
BE-SKAT	2.14	2.25	2.50	2.74	2.72	8.63	8.59	9.65	9.78	10.46	0.05	0.05	0.05	0.05	0.05
BE-BURDEN	2.97	2.26	2.21	4.82	6.01	9.32	8.99	9.72	9.79	9.71	0.04	0.03	0.03	0.06	0.07
**Causal percentage of ~14.6%, PAF = 0.3% (referred to the top row of Fig. 6)**															
ADA	4.13	4.02	4.41	4.78	4.62	7.28	6.73	7.64	8.53	8.44	0.15	0.15	0.14	0.13	0.11
BE-SKAT	3.19	3.47	4.08	4.66	4.71	8.26	8.12	8.68	8.91	9.42	0.07	0.07	0.07	0.07	0.07
BE-BURDEN	4.47	3.80	3.69	7.80	9.32	9.42	9.67	10.06	10.01	10.27	0.06	0.05	0.04	0.09	0.10
**Causal percentage of ~14.6%, PAF = 0.5% (referred to the bottom row of Fig. 6)**															
ADA	4.48	4.24	4.36	4.51	4.58	7.95	7.46	7.87	8.73	8.75	0.14	0.14	0.13	0.11	0.10
BE-SKAT	3.47	3.46	4.17	4.68	4.82	8.70	8.44	8.69	9.48	9.28	0.07	0.07	0.07	0.06	0.06
BE-BURDEN	4.64	3.93	3.71	8.79	10.76	9.99	10.00	10.35	10.01	10.29	0.06	0.05	0.05	0.10	0.12

Supplementary Table S3 Standard deviations of the numbers of true positives and false positives, and positive predictive values (when region length = 20 kb)

5 kb region length	C.V. of no. of true positives			C.V. of no. of false positives			C.V. of positive predictive value		
	r_{isk} =0%	r_{isk} =50%	r_{isk} =100%	r_{isk} =0%	r_{isk} =50%	r_{isk} =100%	r_{isk} =0%	r_{isk} =50%	r_{isk} =100%
**Causal percentage of ~7.3%, PAF = 0.3% (referred to the top row of Fig. 1)**									
ADA	1.49	0.97	0.84	0.86	0.88	0.88	1.52	1.01	0.80
BE-SKAT	0.89	0.69	0.64	0.38	0.40	0.39	0.91	0.73	0.64
BE-BURDEN	0.79	0.66	0.84	0.21	0.21	0.21	0.79	0.65	0.82
**Causal percentage of ~7.3%, PAF = 0.5% (referred to the bottom row of Fig. 1)**									
ADA	1.42	0.72	0.59	0.86	0.87	0.91	1.50	0.77	0.58
BE-SKAT	0.87	0.57	0.51	0.38	0.40	0.41	0.90	0.61	0.52
BE-BURDEN	0.83	0.62	0.87	0.20	0.21	0.21	0.82	0.61	0.85
**Causal percentage of ~14.6%, PAF = 0.3% (referred to the top row of Fig. 4)**									
ADA	1.12	0.79	0.62	0.92	0.93	0.86	0.91	0.62	0.47
BE-SKAT	0.64	0.52	0.48	0.41	0.41	0.43	0.61	0.50	0.44
BE-BURDEN	0.61	0.51	0.63	0.22	0.22	0.23	0.58	0.47	0.59
**Causal percentage of ~14.6%, PAF = 0.5% (referred to the bottom row of Fig. 4)**									
ADA	1.04	0.60	0.46	0.89	0.96	0.82	0.85	0.46	0.34
BE-SKAT	0.59	0.45	0.40	0.39	0.43	0.47	0.53	0.44	0.38
BE-BURDEN	0.59	0.48	0.67	0.23	0.21	0.23	0.57	0.43	0.65

Supplementary Table S4 Coefficients of variation (C.V.) of the numbers of true positives and false positives, and positive predictive values (when region length = 5 kb)

10 kb region length	C.V. of no. of true positives					C.V. of no. of false positives					C.V. of positive predictive value				
r_{isk}	5%	20%	50%	80%	100%	5%	20%	50%	80%	100%	5%	20%	50%	80%	100%
**Causal percentage of ~7.3%, PAF = 0.3% (referred to the top row of Fig. 2)**															
ADA	0.97	0.89	0.73	0.65	0.58	0.73	0.77	0.73	0.75	0.72	0.98	0.91	0.76	0.59	0.55
BE-SKAT	0.58	0.55	0.51	0.48	0.44	0.28	0.30	0.29	0.30	0.30	0.56	0.53	0.49	0.41	0.38
BE-BURDEN	0.58	0.52	0.49	0.64	0.68	0.17	0.16	0.16	0.17	0.18	0.56	0.50	0.47	0.60	0.65
**Causal percentage of ~7.3%, PAF = 0.5% (referred to the bottom row of Fig. 2)**															
ADA	0.80	0.67	0.57	0.45	0.42	0.70	0.68	0.74	0.71	0.65	0.80	0.68	0.57	0.45	0.41
BE-SKAT	0.50	0.45	0.41	0.36	0.33	0.28	0.29	0.30	0.30	0.28	0.50	0.43	0.38	0.34	0.31
BE-BURDEN	0.58	0.48	0.49	0.70	0.77	0.16	0.17	0.16	0.16	0.16	0.56	0.46	0.47	0.66	0.75
**Causal percentage of ~14.6%, PAF = 0.3% (referred to the top row of Fig. 5)**															
ADA	0.87	0.74	0.61	0.50	0.45	0.76	0.76	0.76	0.72	0.62	0.65	0.55	0.43	0.35	0.31
BE-SKAT	0.44	0.42	0.39	0.37	0.34	0.30	0.29	0.32	0.32	0.30	0.39	0.37	0.34	0.29	0.28
BE-BURDEN	0.43	0.41	0.39	0.55	0.52	0.18	0.18	0.17	0.17	0.18	0.42	0.38	0.35	0.48	0.49
**Causal percentage of ~14.6%, PAF = 0.5% (referred to the bottom row of Fig. 5)**															
ADA	0.71	0.62	0.45	0.36	0.32	0.67	0.73	0.74	0.60	0.54	0.52	0.42	0.34	0.27	0.26
BE-SKAT	0.42	0.37	0.35	0.31	0.28	0.29	0.31	0.31	0.30	0.31	0.36	0.33	0.29	0.25	0.24
BE-BURDEN	0.43	0.41	0.38	0.62	0.60	0.19	0.18	0.17	0.17	0.19	0.41	0.39	0.34	0.57	0.57

Supplementary Table S5 Coefficients of variation (C.V.) of the numbers of true positives and false positives, and positive predictive values (when region length = 10 kb)

**20 kb region length**	C.V. of no. of true positives					C.V. of no. of false positives					C.V. of positive predictive value				
r_{isk}	**5%**	**20%**	**50%**	**80%**	**100%**	**5%**	**20%**	**50%**	**80%**	**100%**	**5%**	**20%**	**50%**	**80%**	**100%**
**Causal percentage of ~7.3%, PAF = 0.3% (referred to the top row of Fig. 3)**															
ADA	0.81	0.68	0.60	0.49	0.44	0.60	0.62	0.63	0.60	0.56	0.67	0.58	0.53	0.42	0.38
BE-SKAT	0.45	0.41	0.41	0.36	0.34	0.23	0.23	0.25	0.24	0.25	0.42	0.39	0.36	0.28	0.27
BE-BURDEN	0.45	0.41	0.41	0.57	0.59	0.14	0.15	0.15	0.15	0.15	0.44	0.40	0.38	0.52	0.55
**Causal percentage of ~7.3%, PAF = 0.5% (referred to the bottom row of Fig. 3)**															
ADA	0.72	0.60	0.47	0.35	0.31	0.62	0.63	0.66	0.55	0.51	0.58	0.51	0.42	0.36	0.34
BE-SKAT	0.41	0.38	0.33	0.29	0.26	0.24	0.24	0.25	0.24	0.25	0.38	0.35	0.29	0.24	0.23
BE-BURDEN	0.45	0.40	0.40	0.67	0.67	0.14	0.14	0.15	0.15	0.14	0.43	0.38	0.37	0.63	0.64
**Causal percentage of ~14.6%, PAF = 0.3% (referred to the top row of Fig. 6)**															
ADA	0.69	0.61	0.53	0.39	0.34	0.64	0.61	0.65	0.53	0.47	0.44	0.38	0.32	0.27	0.25
BE-SKAT	0.34	0.34	0.33	0.29	0.28	0.26	0.25	0.26	0.25	0.25	0.31	0.30	0.26	0.23	0.21
BE-BURDEN	0.33	0.33	0.35	0.50	0.51	0.16	0.16	0.17	0.16	0.17	0.31	0.30	0.30	0.45	0.46
**Causal percentage of ~14.6%, PAF = 0.5% (referred to the bottom row of Fig. 6)**															
ADA	0.59	0.52	0.38	0.29	0.27	0.63	0.63	0.59	0.48	0.42	0.37	0.33	0.27	0.23	0.22
BE-SKAT	0.32	0.29	0.27	0.26	0.25	0.27	0.25	0.25	0.25	0.24	0.28	0.26	0.22	0.19	0.18
BE-BURDEN	0.32	0.32	0.35	0.60	0.58	0.17	0.17	0.17	0.16	0.17	0.28	0.29	0.31	0.54	0.54

Supplementary Table S6 Coefficients of variation (C.V.) of the numbers of true positives and false positives, and positive predictive values (when region length = 20 kb)

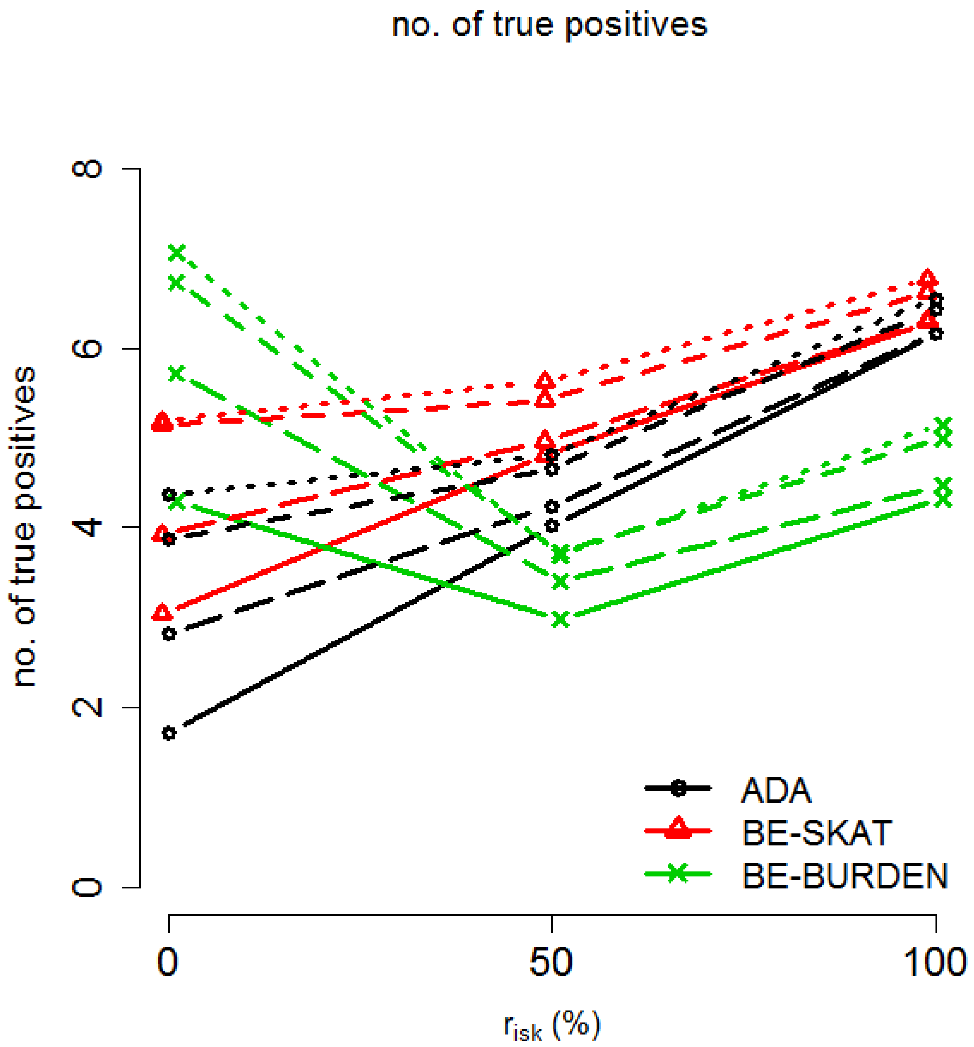

Supplementary Figure S1 Mean numbers of true positives of the three methods (when region length = 5 kb; causal percentage = ~14.6%; PAF = 1%; no. of cases = no. of controls = 500; number of permutations in the ADA test = 10000; number of random subsamples in BE-SKAT and BE-BURDEN = 1000)

Solid line : all results (regardless of the significance of ADA, SKAT, or BURDEN)
Longdash line : results given *P*-values < 0.05 in the corresponding association tests
Dashed line : results given *P*-values < 0.001 in the corresponding association tests
Dotted line : results given *P*-values < 0.0001 in the corresponding association tests

Note: The *x*-axis is the percentage of risk variants from among the total causal variants, whereas the *y*-axis is the mean number of true positives. We here raise the population attributable risk fraction (PAF) of each causal variant to 1% (larger than 0.3% and 0.5% in the main manuscript, Figures 1-6), or the association tests will be hardly significant given a small significance level. We can compare this figure (only the solid lines) with the left column of Fig. 4 in the main manuscript. The PAF associated with each causal variant was enlarged to 1% here. We can see that, as described in the manuscript, as the PAF increased, ADA and BE-SKAT had larger improvements in mean #(*TP*) than BE-BURDEN did.

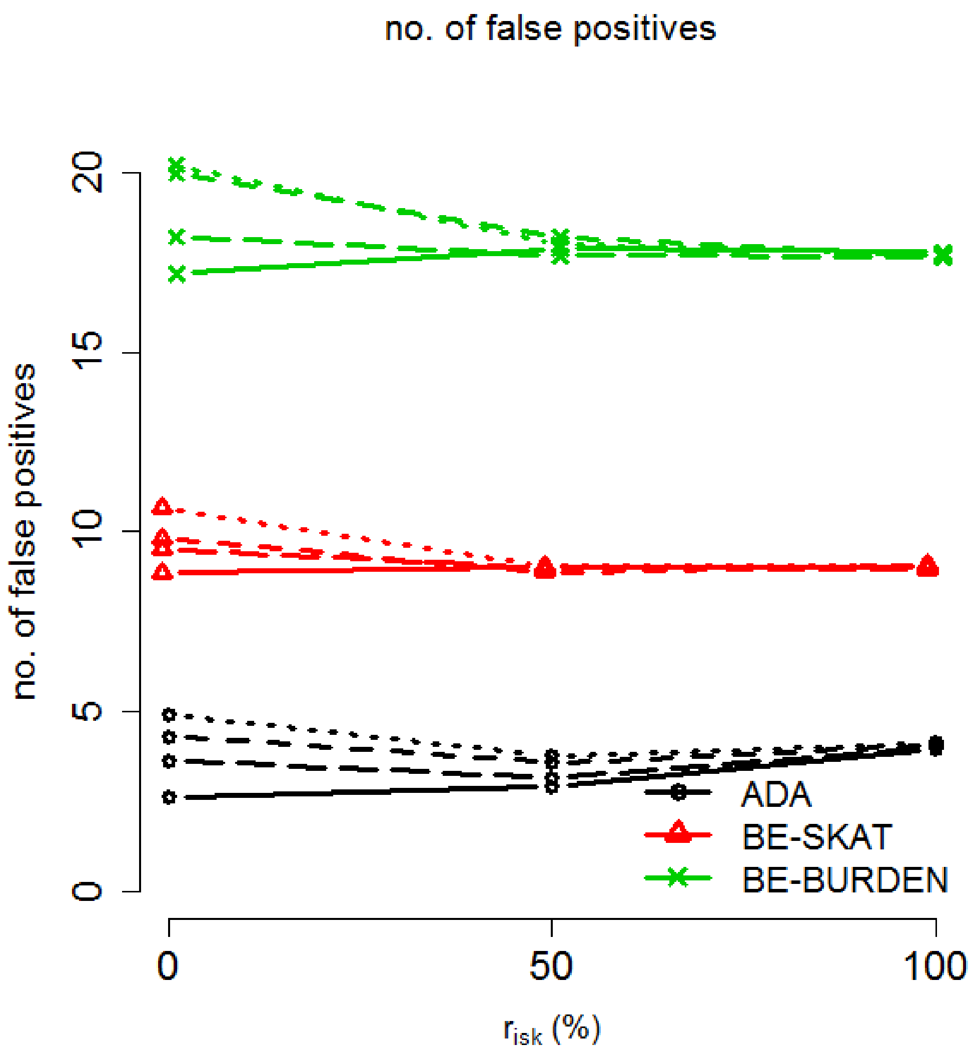

Supplementary Figure S2 Mean numbers of false positives of the three methods (when region length = 5 kb; causal percentage = ~14.6%; PAF = 1%; no. of cases = no. of controls = 500; number of permutations in the ADA test = 10000; number of random subsamples in BE-SKAT and BE-BURDEN = 1000)

Solid line : all results (regardless of the significance of ADA, SKAT, or BURDEN)
Longdash line : results given *P*-values < 0.05 in the corresponding association tests
Dashed line : results given *P*-values < 0.001 in the corresponding association tests
Dotted line : results given *P*-values < 0.0001 in the corresponding association tests

Note: The *x*-axis is the percentage of risk variants from among the total causal variants, whereas the *y*-axis is the mean number of false positives. We here raise the population attributable risk fraction (PAF) of each causal variant to 1% (larger than 0.3% and 0.5% in the main manuscript, Figures 1-6), or the association tests will be hardly significant given a small significance level.

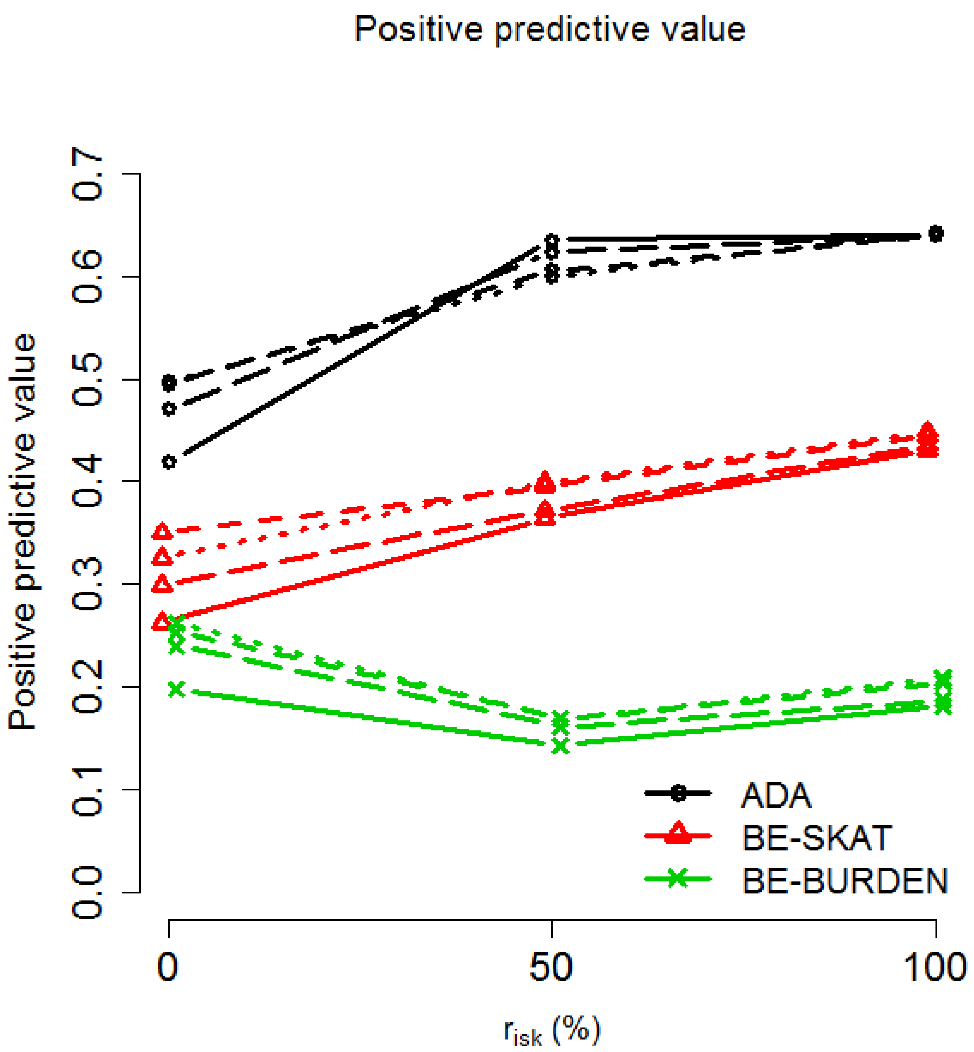

Supplementary Figure S3 Mean positive predictive values of the three methods (when region length = 5 kb; causal percentage = ~14.6%; PAF = 1%; no. of cases = no. of controls = 500; number of permutations in the ADA test = 10000; number of random subsamples in BE-SKAT and BE-BURDEN = 1000)

Solid line : all results (regardless of the significance of ADA, SKAT, or BURDEN)
Longdash line : results given *P*-values < 0.05 in the corresponding association tests
Dashed line : results given *P*-values < 0.001 in the corresponding association tests
Dotted line : results given *P*-values < 0.0001 in the corresponding association tests

Note: The *x*-axis is the percentage of risk variants from among the total causal variants, whereas the *y*-axis is the mean positive predictive values. We here raise the population attributable risk fraction (PAF) of each causal variant to 1% (larger than 0.3% and 0.5% in the main manuscript, Figures 1-6), or the association tests will be hardly significant given a small significance level.
